# Sex-differences in psychosocial sequelae after spontaneous cervical artery dissection

**DOI:** 10.1038/s41598-021-04686-7

**Published:** 2022-01-12

**Authors:** Lukas Mayer-Suess, Moritz Geiger, Benjamin Dejakum, Christian Boehme, Lena M. Domig, Silvia Komarek, Thomas Toell, Stefan Kiechl, Michael Knoflach

**Affiliations:** 1 Department of Neurology, Medical University Innsbruck, Anichstraße 35, 6020 Innsbruck, Austria; 2 VASCage, Research Center on Vascular Ageing and Stroke, Innsbruck, Austria

**Keywords:** Medical research, Neurology

## Abstract

Short- to mid-term functional outcome in spontaneous cervical artery dissection is favorable, but the concomitant psychosocial impact is underreported. We aimed to determine these possible sequelae, with a special focus on sex differences, in our cohort of spontaneous cervical artery dissection subjects. During a standardized prospective in-house follow-up visit we, among other values, evaluated functional outcome (modified Rankin Scale [mRS]), psychosocial measures (return to work-, divorce rate) and health-related quality of life (WHO-QoL-BREF and SF-36-questionnaires). 145 patients participated in the long-term prospective follow-up. Median follow-up time was 6.5 years and excellent functional outcome (mRS ≤ 1) was achieved in 89.0% subjects. 87.6% returned to work and 17.6% married patients had a divorce during follow-up. Even though relevant baseline-/discharge characteristics and functional outcome did not differ between the sexes, women were less likely to return to work compared to men (79.7% vs. 93.8%; P = 0.010) and divorce rate was considerably higher in women (30.2% vs. 9.2%; P = 0.022). Health related quality of life did not differ significantly between the sexes, but women consistently reported lower values. Even though functional outcome is beneficial in most patients, measures to prevent poor psychosocial outcome should be considered in the long-term care of patients with spontaneous cervical artery dissection, especially women.

## Introduction

Functional outcome in spontaneous cervical artery dissection (sCeAD) is considered favorable on a short- and medium term^1–4^. As sCeAD is one of the main reasons for ischemic stroke at a young age, it is not surprising that subjects with all-cause cerebral ischemia before the age of 55 share these generally favorable neurological and functional outcomes^5,6^. Still, psychosocial outcome parameters, such as return to work, depression and anxiety proportions are impaired in all-cause young ischemic stroke survivors^7–14^. Interestingly, even though these data exist in all-cause young ischemic stroke patients, psychosocial outcomes have mostly been neglected in sCeAD^1–4^. The little available data suggest that a sCeAD diagnosis may have profound effect on the psychological state of patients but outcome analyses to date have focused on isolated metrics, such as functional parameters or health-related quality of life, individually^1,15^. Furthermore, analyses on sex-differences in psychosocial outcomes are scarce at best in both patients with all-cause or sCeAD related ischemic stroke^2,15,16^. Therefore, our goal was to provide evidence on long-term psychosocial sequelae after sCeAD within our single center cohort study (ReSect-study) with a special focus on sex differences.

## Methods

### Patient recruitment and selection

Details on the ReSect-Study were published previously^17,18^. In short, electronic medical files of patients treated at the Department of Neurology at the Medical University of Innsbruck due to suspected sCeAD between July 1996 and January 2017 were collected and underwent chart review. Subjects were included if intramural hematoma was evident in T1-weighted fat-saturated MRI imaging of cervical vessels and no timely association to high impact trauma existed. All eligible patients were invited to a standardized in-house clinical follow-up visit at least one year after sCeAD event, which included a physical examination as well as psychosocial outcome assessment through questionnaires and detailed history taking by experienced stroke neurologists.

### Variable definitions

Clinical and functional status of patients were evaluated using the National Institutes of Health Stroke Scale (NIHSS) at hospital admission and the modified Rankin Scale (mRS) at hospital admission,—discharge and follow-up. Excellent functional outcome was defined as mRS ≤ 1. Long-term psychosocial outcome included social reintegration (return to work rate and divorce rate) as well as health related quality of life (HRQoL) measured by WHO Quality of Life-BREF and the 36-Item Short Form Survey (SF 36) questionnaires^19,20^. Return to work was defined as restart of the occupational activity (including stay-at-home parent or caregiver) prior to the index event. The variable was scaled as (1) return to the work in full or reduced capacity (i.e. reduced working hours or in need of assistance for duties previously capable of doing by oneself) or (2) no return to work. The divorce rate was calculated for subjects that were married at baseline.

### Statistical methodology

Descriptive statistical analysis was performed using IBM SPSS Statistics (Version 26.0). Differences in categorical variables were examined by Chi-squared test and in continuous variables by the means of Mann–Whitney-U or T-test. A binomial logistic regression model was created to identify independent risk factors for poor functional and social outcome.

### Standard protocol approvals, registration, and patient consents

This study was approved by the local ethics committee of the Medical University Innsbruck. All patients that took part in the ReSect-study signed appropriate informed consent. All methods were carried out in accordance with relevant guidelines and regulations.

## Results

**Figure 1:**
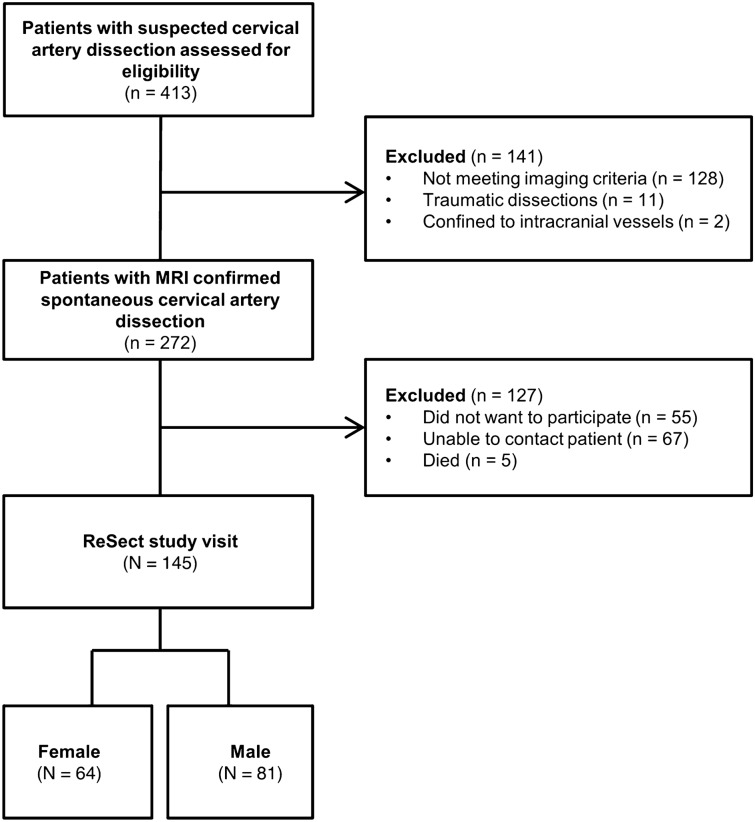
Study flow-chart.

In total, 272 sCeAD patients were eligible for inclusion and 145 of these participated in the in-person follow-up evaluation of the ReSect-Study (Fig. 1). The participating subjects did not differ in baseline characteristics to the excluded subjects (Table 1).

**Table 1:** Differences is baseline characteristics of excluded and included subjects.

	ReSect subjects (n = 145)	Excluded subjects (n = 127)	*P* value
**Baseline**			
*Demographics*			
Age^b^	44.3 (14.1)	45.2 (16.5)	0.75*
Male^a^	81 (55.9)	82 (64.6)	0.14^$^
*Vascular risk factors*			
Diabetes mellitus^a^	2 (1.4)	5 (3.9)	0.18^$^
Hypertension^a^	51 (35.2)	43 (33.9)	0.82^$^
Smoking^a^	43 (29.7)	27 (25.7) (N = 105)	0.11^$^
Preexisting atherosclerotic disease^a^	4 (2.8)	2 (1.6)	0.51^$^
*sCeAD characteristics*			
Anterior circulation^a^	67 (46.2)	63 (49.6)	0.69^$^
Ischemic stroke/TIA^a^	103 (71.0)	93 (73.2)	0.58^$^
*Stroke characteristics*			
NIHSS of 0 at baseline^a^	70 (49.3)	56 (44.4)	0.39^$^
mRS ≤ 1 at baseline^a^	73 (50.3)	56 (44.1)	0.16^$^

sCeAD, spontaneous cervical artery dissection; TIA, transient ischemic attack.

*Mann–Whitney-U test.

^$^Chi^2^-test.

^a^Variables are given as n (%).

^b^Variables are given as median (interquartile range).

**Figure 2:**
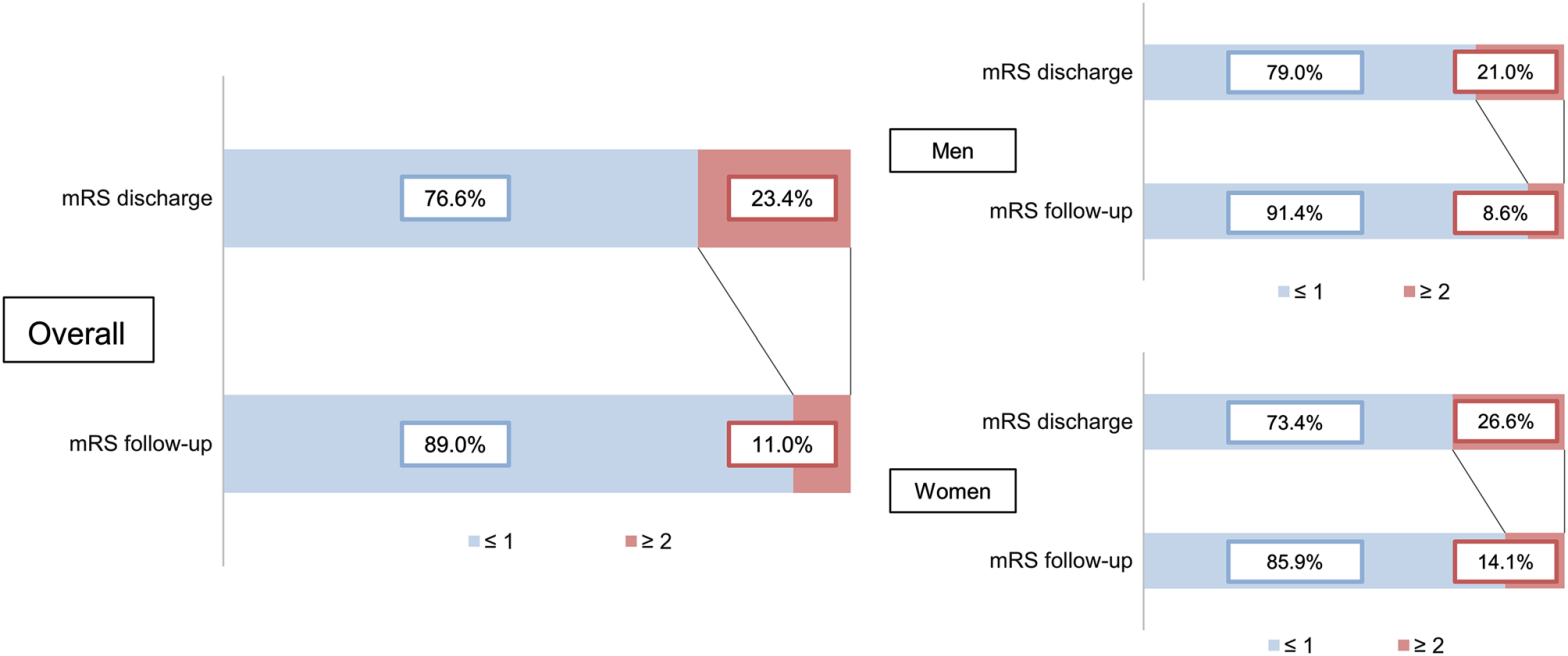
Functional outcome measured by mRS at discharge and follow-up in all ReSect study participants, as well as men and women separately. Men and women did not differ at discharge (P = 0.566^$^) or follow-up (P = 0.301^$^) (^$^Chi^2^-test).

Five sCeAD patients (1.8%), three men and two women, died during the survey period between 1996 and 2017. Demographics, baseline characteristics and follow-up parameters of all ReSect subjects are given in Table 1 with sex differences in baseline and follow-up variables depicted in Table 2. The median age at the initial sCeAD event was 44 (IQR 14.1) years. Of 145 patients, 42 (29%) presented with local symptoms only (headache, Horner’s syndrome, pulsatile tinnitus, cranial nerve palsies). Median time of follow-up was 6.5 years. Excellent functional outcome (mRS ≤ 1) was achieved in 129 (89.0%) patients (Fig. 2).

**Table 2:** Differences in baseline and follow-up characteristics between the sexes.

	Men	Women	P
**Baseline**			
*Demographics*			
Age^b^	47.4 (14.2)	42.6 (12.7)	0.001*
*Vascular risk factors*			
Diabetes mellitus^a^	2 (2.5)	0 (0)	0.26^$^
Hypertension^a^	36 (44.4)	15 (23.4)	0.09^$^
Smoking^a^	21 (25.9)	22 (34.4)	0.33^$^
Preexisting atherosclerotic disease^a^	3 (3.7)	1 (1.6)	0.42^$^
*sCeAD characteristics*			
Anterior circulation^a^	36 (44.4)	31 (48.4)	0.63^$^
Ischemic stroke/TIA^a^	58 (71.6)	45 (70.3)	0.86^$^
*Stroke characteristics*			
NIHSS of 0 at baseline^a^	34 (42.0)	37 (57.8)	0.10^$^
mRS ≤ 1 at baseline^a^	40 (49.4)	36 (56.3)	0.41^$^
**Follow-up**			
*Functional outcome*			
mRS 0 at follow-up	58 (71.6)	45 (70.3)	0.90^$^
*SF 36*			
Physical functioning^c^	92.5 (11.3)	86.3 (21.8)	0.05^-^
Physical role functioning^c^	92.5 (21.4)	87.2 (31.5)	0.30^-^
Emotional role functioning^c^	91.5 (24.7)	89.8 (26.5)	0.72^-^
Fatigue^c^	68.2 (13.6)	64.6 (14.5)	0.18^-^
Emotional well being^c^	79.7 (13.4)	75.6 (17.7)	0.16^-^
Social functioning^c^	92.3 (13.2)	85.7 (23.1)	0.06^-^
Pain^c^	84.3 (20.4)	82.2 (18.7)	0.57^-^
General health^c^	75.1 (16.5)	72.5 (14.9)	0.40^-^
*WHO QoL BREF*			
Physical health^c^	86.5 (11.8)	84.5 (12.5)	0.42^-^
Psychological health^c^	81.5 (11.8)	78.1 (16.3)	0.21^-^
Social relationships^c^	79.4 (16.8)	81.1 (15.5)	0.59^-^
Environment^c^	87.8 (9.4)	87.9 (11.7)	0.98^-^

sCeAD, spontaneous cervical artery dissection; TIA, transient ischemic attack.

*Mann–Whitney-U Test.

^$^Chi^2^-test.

^-^T-test.

^a^Variables are given as n (%).

^b^Variables are given as median (interquartile range).

^c^Variables are given as mean (standard deviation).

**Figure 3:**
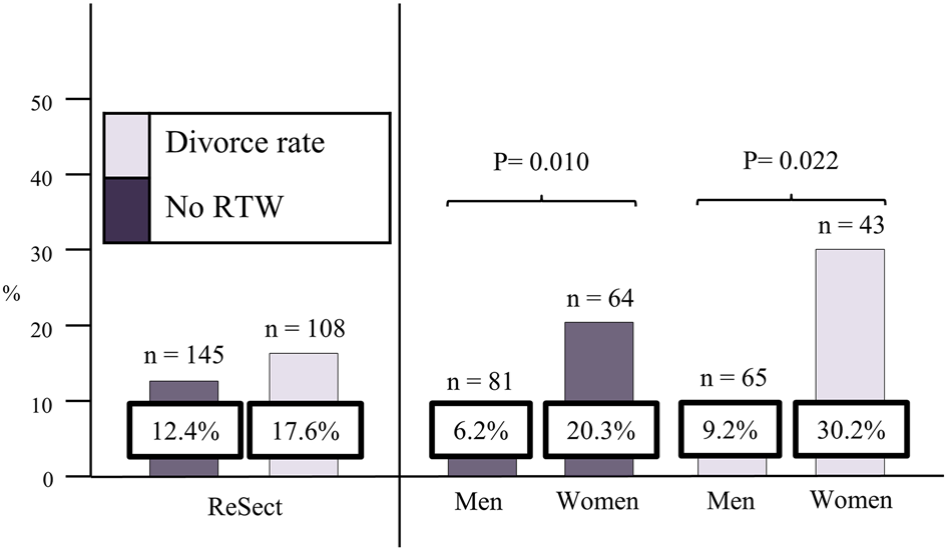
Social reintegration metrics of ReSect-cohort and sex specific subgroup analysis. RTW, return to work.

A total of 127 (87.6%) sCeAD patients had returned to work and 19 of 108 (17.6%) married patients had a divorce during follow-up (Fig. 3).

Males predominated in our cohort and were older than women at initial event (47.4 vs. 42.6 years; P = 0.001). Other baseline characteristics, especially measures of physical functioning at baseline (NIHSS, mRS), as well as functional outcome at discharge (mRS ≤ 1 in men vs. women: 79.0% vs. 73.4%; P = 0.566) and follow-up (mRS ≤ 1 in men vs. women 91.4% vs. 85.9%; P = 0.301) did not differ between sexes (Table 2; Fig. 2).

Still, women were less likely to return to work compared to men (79.7% vs. 93.8%; P = 0.010) and divorce rate after sCeAD was considerably higher in women (30.2% vs. 9.2%; P = 0.022) (Fig. 3). When building a logistic regression model, the variables with borderline significance between the sexes at baseline (P ≤ 0.1: age, hypertension, NIHSS of 0 at baseline) were independent risk factors for poor functional outcome (P = 0.001) but none of them were independent risk factors for poor social outcome. Solely female sex showed significant association with being divorced upon follow-up (P = 0.04, OR 3.055 [CI 1.03–9.00]). HRQoL and its individual components did not differ significantly between the sexes, even though women in general reported lower values of social and physical functioning after sCeAD (Table 2). HRQoL as well as functional and psychosocial outcomes did not differ in sCeAD patients that presented with stroke / TIA or local symptoms only (data not shown).

## Discussion

Even though sCeAD is one of the lead causes of ischemic stroke in young adults, data on long-term outcome, especially on HRQoL and social reintegration, are scarce. Functional outcome is considered favorable in young ischemic stroke patients in general, which extends to individuals with sCeAD. Still, most analyses to date relied on a rather short follow-up time of usually a couple of months or focused on single outcome parameters^8,16,21–26^. Even though functional outcome is beneficial, patient reported outcomes like reduced HRQoL and suboptimal social reintegration after all-cause young ischemic stroke are also important determinants of successful post-stroke recovery on a long run^23,27,28^. To date, little is known about long-term psychosocial sequelae of sCeAD. We present data from a single-center long-term cohort combining functional with psychosocial patient reported outcomes. In general, our study confirms that the beneficial short-term functional outcome after sCeAD persists in the long-run as excellent functional outcome (mRS ≤ 1) was evident in 90% of patients after a median follow-up of 6.5 years. Social reintegration parameters overall were in line with the functional metrics as almost 90% of patients returned to work. This is much higher than the 60% reported for all-cause young ischemic stroke survivors^11–13^. Furthermore, only one in six subjects had a divorce during the follow-up period. Even though literature to date reports equal outcomes in sexes after sCeAD^1,27,29–33^, we observed a pronounced gap between men and women with females being significantly less likely to return to work and more likely to get divorced after sCeAD (Fig. 3). Differences between the sexes in psychosocial measures and outcomes have been investigated in detail in other vascular diseases, such as coronary artery disease^34–39^. As psychological factors have a negative impact on outcome of ischemic heart disease, it is tempting to speculate that the same might be true for cerebrovascular diseases, especially in young patients^40,41^.

The little comparison concerning divorce rate that literature offers describes a range from 50% in rather small case studies of coronary heart disease and a 3 years 7% divorce rate in all cause young ischemic stroke subjects^16,42^. In both instances, no sex differences had been reported. But, as divorce itself has been linked to an increased risk of acute myocardial infarction, especially in women, the sex differences with women more frequently getting divorced after sCeAD has to be taken seriously^43^. The reasons for the sex differences in our study remain unclear. One might speculate on a gap in psychosocial safety-nets, similar to coronary artery disease patients^37^, or sex specific demands in social functioning. Previous studies have demonstrated that women are more likely to be discharged to assisted living facilities compared to men in general ischemic stroke cohorts^44,45^. Yet, this can be explained by a worse pre-stroke physical functioning in women due to older age at stroke onset and more severe strokes, which was not apparent in our current analysis of sCeAD patients.

Strengths of this study are the stringent inclusion criteria, providing a cohort of patients with a definite sCeAD diagnosis. Additionally, data of our well-characterized single-centre cohort rely on long-term standardized in-person follow-up.

Limitations include that not all subjects completed the HRQoL questionnaires. Even though baseline and follow-up characteristics between those assessed within the clinical visits and those evaluated via chart review did not differ (Table 1), we cannot completely rule out a selection bias as patients with low HRQoL and/or depression may be reluctant to participate in a clinical study, as has been described previously on multiple occasions in studies investigating depression^46,47^. Reasons on why subjects did not want to attend the in-house study visit were not routinely assessed during the recruitment phase of the study. Furthermore, we did not record the timing of social reintegration values (divorce and return to work) after sCeAD and had information on divorce proportions but not on other outcomes in long-term unmarried cohabitating relationships. Lastly, it would have been rewarding to present a control group of subjects suffering from other cause ischemic stroke.

In conclusion, even though functional outcome is beneficial in most sCeAD patients there is a need for measures to prevent poor psychosocial outcome, especially in women.

## Data Availability

The datasets generated during and/or analysed during the current study are available from the corresponding author on reasonable request.
